# Supplementary figures and images for: Optimal Contact Position of Subthalamic Nucleus Deep Brain Stimulation for Reducing Restless Legs Syndrome in Parkinson’s Disease Patients: One-Year Follow-Up with 33 Patients
Source: Brain Sci. 2022 Dec 1;12(12):1645. doi: 10.3390/brainsci12121645 (PMC9775276; doi:10.3390/brainsci12121645)

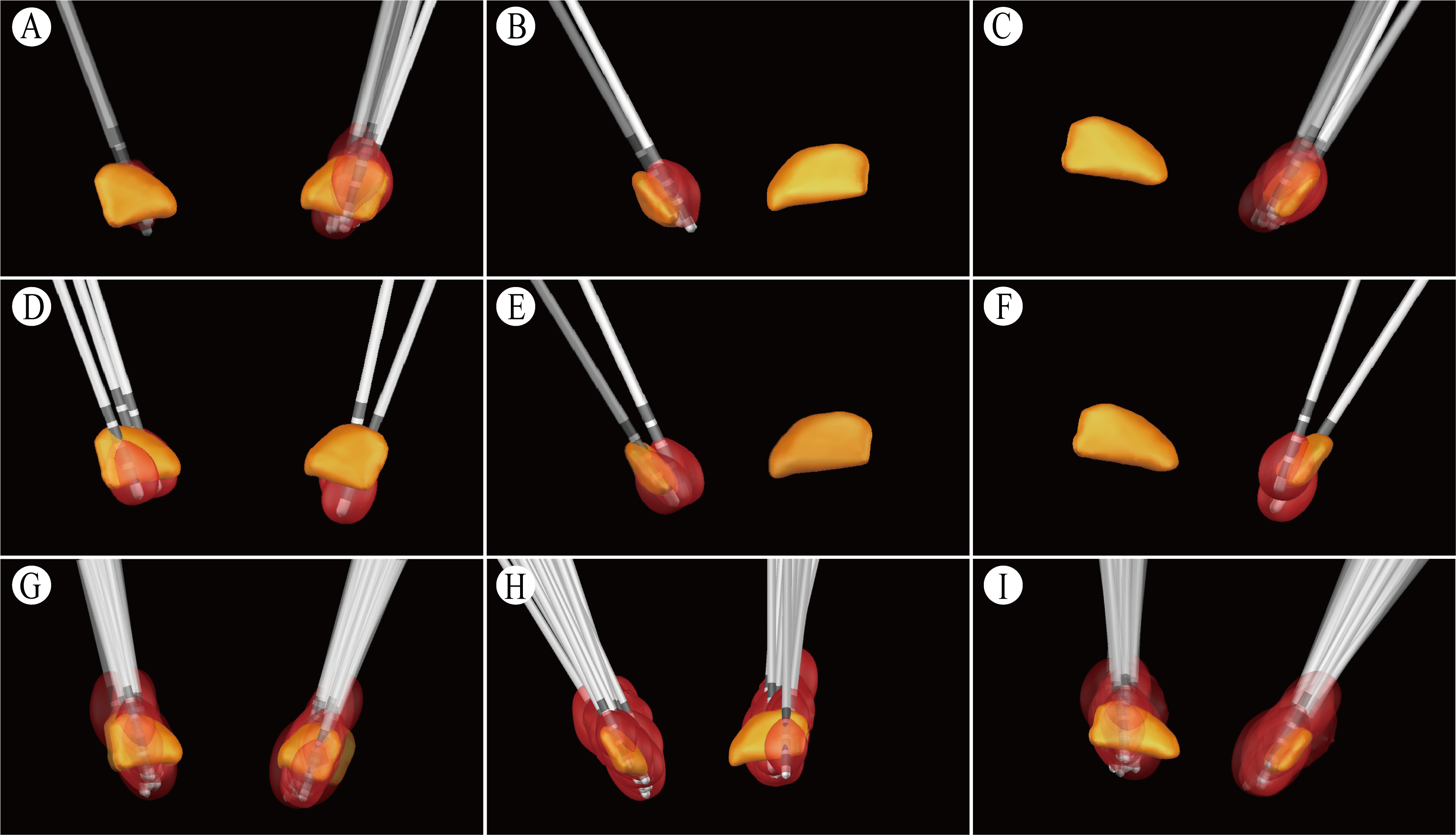

Supplement: Supplementary file 1 [file brainsci-12-01645-s001.zip › brainsci-1928553-supplementary.tif]
